# Predictors of Adolescent Depressive Symptoms

**DOI:** 10.3390/ijerph18094508

**Published:** 2021-04-23

**Authors:** Vilija Malinauskiene, Romualdas Malinauskas

**Affiliations:** 1 Department of Population Studies, Institute of Cardiology, Lithuanian University of Health Sciences, Sukileliu 17, 50157 Kaunas, Lithuania; vilija.malinauskiene@gmail.com; 2 Department of Physical and Social Education, Lithuanian Sports University, Sporto 6, 44221 Kaunas, Lithuania

**Keywords:** adolescents, depressive symptoms, sense of coherence, hierarchical linear regression analysis, predictor

## Abstract

The present study expands the existing literature and supplements today’s knowledge on the relationship between personal, psychosocial and lifestyle factors and depressive symptoms among adolescents. The study aimed to investigate the variety of depressive symptoms predictors—personal resources, adverse school and family, health, lifestyle-related (sense of coherence, self-esteem, school involvement, negative acts at school, family stress and violence, psychosomatic health complaints, physical activity, smoking, alcohol) as well as gender, employing hierarchical linear regression analysis in a large representative sample of adolescents (*N* = 2212) in Kaunas, Lithuania. Four blocks of predictors were employed in hierarchical linear regression analysis. In the final model 64.9% of depressive symptoms were explained by all the predictors. Sense of coherence was the strongest predictor of depressive symptoms (standardized regression coefficient β = −605, *p* < 0.001 in the first model and β = −263, *p* < 0.001 in the final model after adjustment for all other independent variables) and accounted for 36.6% of variance. In conclusion, this study supports the notion that depressive symptoms among adolescents have multifactorial origins with many predictors showing significant effect seizes. Therefore, high sense of coherence and self-esteem, school involvement, higher levels of physical activity would be protective and influence lower levels of depressive symptoms among adolescents. Exposure to negative acts at school and negative experiences in the family, psychosomatic health complaints, smoking would increase the probability of depressive symptoms. Girls are more prone to depression as compared to boys.

## 1. Introduction

Depression is a serious and common public health problem that poses a heightened risk in adolescence. Meta-analyses show a rather stable trend of adolescent mental health complaints and depressive symptoms internationally in non-clinical populations between the 1970s and 2010s [1]. Worldwide, the prevalence of depressive disorders in childhood and adolescence is 4–5% [2]. The main symptoms include difficulties in concentrating, lack of self-worth, low mood, joylessness, a loss of activities and interests, social withdrawal, giving up leisure activities, changes in appetite, sleep disruption, and—in moderate to severe forms—suicidal thoughts and acts.

The issue of the current system is that depression cannot be related to one or few factors, explaining the origin of the disorder. In short, according to the different theories, depression may be due to (1) biological reasons; (2) insecure attachment; (3) lack of reinforcement of previously reinforced behaviors; (4) negative interpersonal relations and relations with one’s environment and the resulting negative consequences; (5) attributions made by individuals about themselves, the world and their future; and (6) sociocultural changes. It is likely that no single theory can fully explain the genesis and persistence of depression, although, currently, negative interpersonal relations and relations with one’s environment and sociocultural changes (economic, political, and demographic), lack of internal resources may explain the observed increase in the prevalence of depression. As there are many theories and many predisposing factors explaining adolescents’ depression, there is a need for an integrated approach, capturing different sides of the phenomenon, involving epidemiological research methods with complex statistical methods allowing simultaneous disclosure of the effect of many predisposing factors or predictors on the occurrence of depressive symptoms among adolescents.

The origin of depression is multifactorial, and risk and protection factors may be found not only in the school environment, in the family and social contexts, but in psychological surrounding and lifestyle as well.

The sense of coherence (SOC) is a resource that makes people manage tension, to reflect about their internal and external resources, to identify and mobilize resources, to promote effective coping by finding solutions, and to resolve tension in a health-promoting manner [3]. Internal resources refer to personal characteristics which influence individual development and is defined as the extent to which one has a pervasive, enduring and dynamic feeling of confidence. Studies confirm that SOC is associated with mental health problems. Ericsson and Lindstrom, 2006, found that feeling depressed was significantly associated with low SOC among adolescents [4]. The findings from a recent study provided support for the significant role of SOC as a coping resource, especially in relation to adolescents’ mental health and supported a compensatory role of SOC on the association between stress and health during adolescence [5]. In order to promote positive functioning, health and wellbeing in the adolescent population, it is important to gain a better understanding of how SOC relates to adolescents’ overall experience of good mental health, as well as investigating the role of potential harmful factors in this context. Among internal factors, self-esteem should be another important source of resilience. Although there are many definitions and different types of self-esteem in psychology, unless stated otherwise, self-esteem is usually defined as a set of one’s own thoughts and feelings about his or her worth and importance [6] or the general evaluation and appraisal of one’s worth [7]. Self-esteem refers to positive or negative self-evaluations [6], and low self-esteem strongly predicted depression [8]. Studies have shown that high self-esteem acts as a buffer under stress, hence reducing harmful effects of stress on depression in youth [9]. Meanwhile, few researchers have investigated the potential protective role of self-esteem in the development of psychiatric problems in adolescence. Hence, the role of self-esteem in the development of psychiatric conditions is largely unknown; therefore, we included self-esteem as a predictive variable for depressive symptoms among others.

Adolescent depression was also associated with an increased risk of other adversities in adulthood, including adverse psychosocial factors at school and in the family, low educational attainment and problems related to interpersonal relationships [10]. Low school connectedness increases the risk for depressive symptoms. A close social bond with fellow students further reduces the risk of developing mental health problems [11]. The study investigating the association of school bullying experiences with depressive symptoms confirmed that bullying victimization was associated with feeling depressed (odds ratio 2.61) [12]. Therefore, we included exposure to negative acts at school and school involvement as possible predictive psychosocial variables for depressive symptoms in our study.

Recent studies provide important evidence of the link between child maltreatment and depression and highlight the particularly larger association with emotional maltreatment in childhood [13,14]. A systematic review and meta-analysis has demonstrated a robust association between child maltreatment and the development of mental disorders [15]. The awareness of the serious long-term consequences of child maltreatment should encourage effective interventions to protect children from violence [16]. Taking into account the possible associations of depressive symptoms with family stress and violence, we chose to investigate this variable as predictive.

It is evident that subjective health complaints are associated with depressive symptoms among adolescents. Research indicates that high depressive symptoms were associated with poor self-rated health [17] and psychosomatic health complaints [18]; therefore, it was interesting to reveal the impact of psychosomatic health complaints in the general predictive model of depressive symptoms.

Lifestyle factors may be also linked to depressive symptoms among adolescents. The relationship between screen-based sedentary behaviors and symptoms of depression has been established [19] and those who were active in sports were significantly less likely to report symptoms of depression [20]. Research findings suggest that moderate-intensity exercise may be an optimal intensity of exercise for the promotion of mental health [21]. Smoking has been linked with depressive symptoms in adolescents as well [22]. Higher levels of depression symptoms were associated with earlier onset of alcohol use, more frequent consumption and intoxications [23]. Lifestyle habits usually serve as confounders in epidemiological studies, therefore they were included in our study as well.

In the majority of research studies on adolescents’ depressive symptoms only several predictors have been investigated. Therefore, 10 independent variables were selected in this study. From a health promotion perspective, more knowledge of how various psychosocial factors, internal resources, lifestyle habits are related to adolescent depressive symptoms is needed. In order to gain more knowledge of which of these factors future interventions among school-based populations of adolescents should prioritize, there is need to simultaneously investigate the impact of these factors on depressive symptoms. Investigating such associations could inform practice and policy and would help to promote adolescents’ mental health. Taking into account that the origin of depressive symptoms among adolescents is multifactorial, we investigated the variety of predisposing personal, school and family, health, lifestyle related factors (sense of coherence, self-esteem, school involvement, negative acts at school, family stress and violence, psychosomatic health complaints, lifestyle: physical activity, smoking, alcohol, as well as gender) employing hierarchical linear regression analysis in a large representative sample of adolescents. The aim of the study was to reveal the most important and significant in the sense of size effects predictors of depressive symptoms among adolescents. Based on theory and earlier research, we hypothesized that there is a positive association between depressive symptoms and exposure to negative acts at school and family stress and violence, low self-esteem and psychosomatic health complaints and lifestyle habits and, probably negative associations with sense of coherence, school involvement. In accordance with other studies, we hypothesized that girls are more prone to depressive symptoms as compared to boys.

## 2. Materials and Methods

### 2.1. Study Design

The study was planned like cross-sectional survey among adolescents.

### 2.2. Participants, Procedure and Measures

This cross-sectional survey included adolescents from 16 secondary schools in Kaunas (the second largest city in Lithuania). Schools were randomly selected from the institutional registry list of the Ministry of Education and Science. The study was approved by the Kaunas municipality Education Department, Regional Biomedical Research Ethics Committee (ethics code Nº BEC-SP(M)-88). Data were collected from November to December 2018 by using self-administered questionnaire. All pupils and parents received written information about the purpose of the study and those with a written permission from the parents participated. This sample represents the Kaunas city, the second largest city in Lithuania. In a total 60 5th–8th grade classes, 2474 pupils were selected and 2212 fully completed questionnaire (response rate 89.4%). 49.6% girls and 50.4% boys comprised the sample. The average age was 13.09 years; SD—1.56

The survey assessed the following variables: pediatric depressive symptoms, self-esteem, sense of coherence, psychosomatic health complaints, leisure physical activity, smoking levels, alcohol consumption, family stress, school involvement, and exposure to negative acts.

### 2.3. Pediatric Depressive Symptoms

Pediatric Depressive Symptoms were assessed with a short from of the Patient-Reported Outcomes Measurement Information System (PROMIS) scale which consists of 8 items [24]. Adolescents are asked to consider their feelings over the past 7 days. Sample items include: “I felt sad”, “I felt lonely”, and “It was hard for me to have fun”, etc. Response choices are Never, Almost never, Sometimes, Often, and Almost always. A total score was computed by summing up all the items; the higher scores reflect greater levels of depressive symptoms. This scale was selected based on its relative brevity, appropriateness for use with a wide age range of youth, promising evidence of reliability and validity, and the availability of normative data that can be used to help gauge the severity of a responding youth’s depressive symptoms [25] and was adapted in the Lithuanian adolescent population [26]. In the present study, the internal consistency of the scale tested by Cronbach’s alpha was 0.814.

### 2.4. Self-Esteem

Self-esteem was evaluated using the Rosenberg scale [27]. This scale includes 10 questions with four possible answers: strongly agree, agree, disagree and strongly disagree, which were then scored with 1, 2, 3 and 4 points, respectively. Cultural adaptation of the scale was performed [26]. Five negatively worded items were reverse-scored (4–1). Total score was counted after adding the points corresponding to all the items in the scale (in a range between 10 and 40 points in our study), the higher scores indicating lower self-esteem. The most common practice in scientific studies is using the mean of global (total) scores as the main criterion for interpreting the results of the application of the Rosenberg Self-esteem scale [6,28]. In this study, the results showed a good internal consistency of the scale, evaluated by Cronbach’s alpha coefficient, which was 0.81.

### 2.5. Sense of Coherence

The SOC-13 includes items of the main aspects of sense of coherence: comprehensibility, manageability, and meaningfulness [29]. Participants selected from a 5-point Likert type scale a response to each of the items, ranging from 1 to 5. Antonovsky [30] stressed the holistic nature of the SOC scale and, consequently, a sum score is commonly used. In this study, the total score of the SOC-13 was calculated by summing the responses to each of the items and ranged from 13 to 65 in this study, with a higher score indicating the stronger sense of coherence. Lithuanian cultural adaptation of the scale was performed previously [31] and showed good psychometric properties. In this study, the SOC-13 Cronbach’s alpha was 0.894.

### 2.6. Psychosomatic Health Complaints

Psychosomatic health complaints were assessed using the questions from the HBSC study [32]. Respondents were asked how often they had experienced the following psychosomatic symptoms in the last 6 months: headache, stomach ache, back pain, “feeling dizzy”, “feeling tense”, sleeplessness, “get up tired in the morning”. Response options for each symptom were (1 = “rarely or never”, 2 = “about every month”, 3 = “about every week”, 4 = “more than once a week”, 5 = “about every day”). The answers scores were summarized to represent scores of psychosomatic complaints. The higher scores indicate greater symptoms of psychosomatic health complaints. In this study, the internal consistency measured by Cronbach’s alpha was 0.885.

### 2.7. Leisure Physical Activity

Leisure physical activity was assessed by a single question as proposed by WHO [33]: “How often in leisure time you have been physically active (sports, running, etc.), no less than 60 min in the way that your breathing becomes hard and sweat appears” with possible seven answers “every day”, “4–6 times per week”, “2–3 time per week”, “once a week”, 2–3 time per month”, “few time per year and less often”, “unable to exercise due to illness” with higher scores indicating lower levels of physical activity.

### 2.8. Smoking Levels

Health behaviors were evaluated by methods in the HBSC study [32]. Smoking levels were assessed using a single item: “At this moment, how often do you smoke?”. The responses options were: “I have never smoked” = 1, “I smoked some time before, but I quit” = 2, “I occasionally smoke” = 3, “I smoke at least one cigarette a day” = 4, higher scores indicating lower levels of smoking.

### 2.9. Alcohol Consumption

Students were asked to indicate their alcohol consumption (HBSC study, [32]) with 5 possible answers: “I do not drink at all”, “I drink 2–3 times a year”, “I drink occasionally”, “I drink each month”, “I drink once a week or more frequently”, higher scores indicating higher levels of alcohol consumption.

### 2.10. Family Stress

Psychosocial factors at school and in the family were assessed by the HBSC study methods. Family stress was assessed [32], using a single question: “do you feel calm and satisfied at home?” with 4 possible answers: “I feel calm at home”, “I experience rare conflicts in the family”, “Often conflicts in the family”, “permanent tension in the family”. Domestic violence was assessed by single question: “Do you sustain violence in your family (beating, mocking, humiliation, threat)?” with possible answers: “never”, “experienced 1–2 times in my life”, “experience 2–3 times a month”, “experience once a week”, “experience several times per week”. The answers to family stress and violence were summed to create a continuous variable with higher scores indicating higher levels of family stress and violence.

### 2.11. School Involvement

School involvement was assessed (HBSC study, [32]) by summing up the answers to a few questions; the higher the score, the better the school involvement: “How do you feel at school?” with five possible answers indicating a better school climate and “How many friends have you at school” with five possible answers from “no one friend” to “six and more friends”.

### 2.12. Exposure to Negative Acts

Exposure to negative acts at school was evaluated by internationally accepted Olweus Negative acts questionnaire [34] using a set of questions on different forms of bullying that students might have experienced in the past couple of months (verbal bullying, social exclusion or isolation, physical bullying, bullying through lies and false rumors, having money or other things taken or damaged, threats or being forced to do things, sexual bullying, etc.) with five possible answers: “it hasn’t happened to me in the past couple of months”, “only once or twice”, “2 or 3 times a month”, about once a week”, “several times a week”. The total score was calculated by summing up all the answers and creating the continuous variable of exposure to negative acts at school. In the present study, the internal consistency of the questionnaire tested by Cronbach’s alpha was 0.810.

### 2.13. Statistical Analysis

We used SPSS 24.0 (IBM, Armonk, NY, USA) in the statistical analysis. The variables were checked for normality, then Pearson correlations were calculated for the study variables. Firstly, simple linear regression analysis was used for each predictor to check for significance and effect seizes expressed in regression coefficients. Then, hierarchical regression analysis was performed with depression symptoms as dependent variable and three blocks of predictors. The first block included sense of coherence as it was the strongest predictor in simple linear regression. In the second block, negative experiences (negative acts at school and family stress and violence) were added, the third block was supplemented with three social variables (self-esteem, psychosomatic health complaints and school involvement). Finally, gender and lifestyle (physical activity, smoking and alcohol) were included additionally into block four. To illustrate change in effect sizes when controlling for various blocks of variables, we report regression coefficients (standardized β). Explained variance was evaluated by R square and R square change. Statistical significance was set at a *p*-value of less than 0.05.

## 3. Results

### 3.1. Descriptive and Correlation Analyses

**Figure 1:**
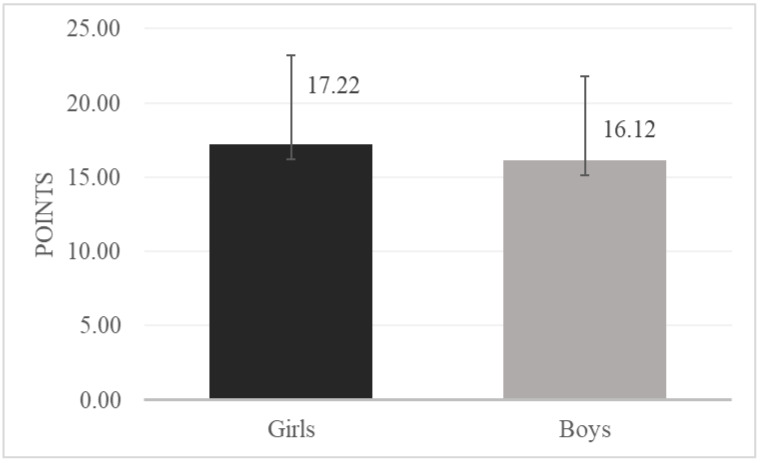
Means and standard deviations of depressive symptoms among girls and boys.

Depressive symptoms were significantly more prevalent among girls as compared to boys (*t* (2126) = 4.37; *p* < 0.001) (Figure 1).

We calculated correlations between study variables (Table 1). Depressive symptoms correlated significantly with all study variables; the highest correlation was found with sense of coherence (−0.612, *p* < 0.001), the higher the sense of coherence, the lower the levels of depressive symptoms. A positive correlation of depressive symptoms was found with psychosomatic health complaints (0.578, *p* < 0.001), negative acts at school (0.530, *p* < 0.001), family stress and violence (0.405, *p* < 0.001), lower self-esteem (0.467, *p* < 0.001), lower physical activity (0.216, *p* < 0.001), smoking (0.078, *p* < 0.001), alcohol (0.082, *p* < 0.001). Negative correlations were found with school involvement (−0.442, *p* < 0.001), more depressive symptoms with less school involvement.

### 3.2. Regression Analyses

Table 2 shows the strength of crude and adjusted associations from simple and hierarchical linear regression analyses between the covariates and the dependent variable (depressive symptoms) described in terms of effect sizes (standardized β) and explained variance (R square). In a simple linear regression analysis, SOC was the strongest predictor of depressive symptoms with standardized β = −0.605 and R square 0.366. A strong positive relation was found between depressive symptoms and negative acts at school as well as psychosomatic health complaints. All predictors showed a significant effect (*p* < 0.001) on depressive symptoms with lowest standardized β for lifestyle (physical activity, smoking, alcohol and gender).

We performed hierarchical linear regression analysis including four blocks of predictors for depressive symptoms as a dependent variable among adolescents. Sense of coherence was the strongest predictor of depressive symptoms and accounted for 36.6% of variance in Model I. Secondly, in Model II negative experiences at school and in the family were included (negative acts at school, family stress and violence) and this model accounted for 13.5% additional variation of the previous model. R square change from Model I to Model II was statistically significant, *p* < 0.001. Then, the third block of predictors was included into Model III (self-esteem, psychosomatic health complaints and school involvement) and contributed to a considerable increase in the explained variance (13.2%; *p* < 0.001) as compared to Model II. Finally, in Model IV the fourth block of predictors was included (gender and lifestyle, e.g., physical activity, smoking and alcohol), adding some predicting power in hierarchical regression with significant increase in R square change (1.7%; *p* < 0.001). In the final Model IV 64.9% of depressive symptoms were explained by all the predictors. When all variables were added into model IV, psychosomatic health complaints and SOC revealed the largest effect sizes, though the impact of all the predictors remained statistically significant (*p* < 0.001), except alcohol (*p* = 0.328). Thus, lower sense of coherence, more negative acts at school and family stress and violence, lower self-esteem and school involvement, more psychosomatic health complaints, gender (being a girl was positively associated with depressive symptoms), lower levels of physical activity and higher levels of smoking predicted more depressive symptoms among adolescents.

## 4. Discussion

To date, in epidemiology, it remains unknown which factors increase the risk for developing mental health problems and which factors are protective and help children and adolescents to grow up mentally healthy.

In this study, we investigated the variety of depressive symptoms predictors (personal, school and family, health, lifestyle related) among adolescents. Our results indicated that sense of coherence and psychosomatic health complaints were the strongest predictors when controlling for all investigated risk factors. Though in the final model effect sizes of SOC and psychosomatic health complaints diminished almost two-fold (from β = −0.605, *p* < 0.001 to β = −0.263, *p* < 0.001 for SOC and from β = 0.575, *p* < 0.001 to β = 0.297, *p* < 0.001 for psychosomatic health complaints), their effect remained statistically significant. Our finding coincides with other research studies. Moksnes et al. (2012) [35] analyzed data from 1209 adolescents in Mid-Norway and found that adolescents with a strong SOC exhibited lower levels of depression and that the associations found give support for the implications of salutogenic factors in relation to emotional health in adolescents. Another study found inverse associations between PTS symptoms and strong SOC and supported the idea that SOC is strongly related to mental health [36]. Additionally, the study using analysis of structural equation modeling found that SES was associated with adolescent depressive symptoms indirectly through maternal care separately, as well as through maternal care and adolescent SOC sequentially [37]. Furthermore, a recent study found that adolescents with a weaker sense of coherence and a tendency toward depression were much more likely to become addicted to energy drinks, while a strong sense of coherence diminishes the effects of depression [20]. Mental health complaints have also shown to commonly co-occur and highly correlate with somatic health complaints (reoccurring pains and aches) in adolescent population studies [1]. Therefore, the combination of mental health complaints and somatic health complaints are often considered to be unidimensional based on empirical and theoretical grounds. The study investigating the associations between psychosomatic health complaints and depressive symptoms found that the best predictor of a positive screen for depressive symptoms, maximizing both sensitivity and specificity, was the presence of more than three health symptoms [18].

Sense of coherence was the strongest predictor of depressive symptoms and accounted for 36.6% of variance in a simple linear regression. Then, we employed four blocks of predictors into the hierarchical linear regression (adverse school and family experiences, psychosocial factors, lifestyle) and every block increased the predicting power of the model and in the final Model 64.9% of depressive symptoms was explained by all the predictors. Exposure to negative acts at school and family stress and violence were significant predictors of depressive symptoms. Other studies confirmed our results indicating that victims of school bullying experienced adverse emotional consequences and depression [12]. Maltreatment during childhood was linked to12-month diagnoses of major depressive disorder [14].

Self-esteem and school involvement remained significant predictors of depressive symptoms when controlling for all other investigated factors. Other studies confirm our findings indicating that low self-esteem strongly predicted depression [8]. A study, based on 3-year longitudinal data on adolescents with psychiatric problems, showed that high self-esteem at baseline predicted fewer symptoms of depression 3 years later after controlling for prior symptom levels, gender, therapy (or not), and medication [37]. Self-esteem is a critical internal source of resilience of adolescents. Self-esteem serves an anxiety-buffering function. In this sense, self-esteem protects individuals from anxiety and thereby contributes to their positive development in terms of cognition and emotion, subjective well-being and mental health and social behavior [38,39].

Lower school involvement has been found to increase the probability of depressive symptoms among adolescents [11,40] and the recovery from depression could be achieved by encouraging a healthy lifestyle, enhancing social skills among adolescents [41].

It is interesting to note that the predictive power of lifestyle factors was low, though significant with effect sizes expressed in regression coefficients from β = 0.088, *p* < 0.001 for physical activity to β = 0.050, *p* < 0.001 for smoking. Alcohol lost statistical significance in the final model. Literatures sources indicate that symptoms of depression were frequent in adolescents and were associated with unhealthy lifestyle factors such as low physical activity [42]. Other research finding suggest that exercise may help to mitigate symptoms of depression by reducing inflammation [21] and those adolescents that were active in sports were significantly less likely to report symptoms of depression [20]. We investigated gender differences in depressive symptoms among adolescents and found that girls are affected twice as often as boys (β = −0.056, *p* < 0.001) and our findings coincide with literature data [43].

The contribution of this study in the area of adolescents’ depressive symptoms research is based on the attempt to capture various depressive symptoms predictors originating from psychosocial environment, internal resources, lifestyle and subjective health. The study confirmed the importance of sense of coherence, firstly, as the main predictor of adolescents’ depressive symptoms. Then, psychosocial factors at school and in the family and indicators of self-involvement and self-value and subjective health are no less important. Additionally, lifestyle habits show a complementary, but not so great effect in the associations with adolescents’ depressive symptoms.

## 5. Strengths and Limitations

The major strengths of the current study are the large sample size and the precision of the estimated associations, which make chance an unlikely explanation for the observed results. An entire population was included, and the participation rate was high, reducing the possibility of selection bias.

One limitation of our study was that we used self-reported data and our measure of depression was non-diagnostic which can be prone to recall bias. We did not use a diagnostic tool or interview to evaluate mental health problems. However, the eight-item Pediatric Depressive Symptoms scale indicated meaningful results in previous studies [24,25].

Another limitation is that the cross-sectional design limits suggestions about the direction of causality between psychosocial, lifestyle and resilience factors and symptoms of depression. Despite the cross-sectional study design, our findings indicate that a possible direction for future work may be to explore intervention strategies that are designed to increase self-esteem and strengthen SOC, school involvement, especially among girls with psychosomatic health complaints. Although we proposed the directional influences of predictors on depressive symptoms, the current study’s cross-sectional nature has precluded us from making a causal inference about these constructs. Longitudinal design would permit the examination of the reciprocal and bidirectional associations between depressive symptoms and associated variables.

The results of this study may be regarded as representative of Kaunas city adolescents, the second largest city in Lithuania and cannot be generalized to wider populations. Nevertheless, the present results from Kaunas secondary school adolescents are important for planning purposes. Future research studies could overcome those limitations with increasing geographical area of the investigated adolescents’ population. Lastly, this study did not collect data about the participants’ (or their parents’) socioeconomic status, which might be related to adolescents’ development outcomes (e.g., the higher socioeconomic status is, the higher SOC and self-esteem may be), though we did not think it necessarily influence the associations among the outcomes (e.g., it does not necessarily moderate the association between SOC, self-esteem and depressive symptoms). This demographic factor should be considered in future studies. Further research should aim to investigate more harmful or protective factors attributed to depressive symptoms among adolescents, including socioeconomic status, academic performance, traumatic life events, type of parenting, parents’ depression, computer and computer games addiction, and, probably, environmental factors as ambient air and noise pollution.

## 6. Conclusions

In conclusion, this cross-sectional study among 5th–8th grade students supports the notion that depressive symptoms among adolescents have multifactorial origin with many predictors (personal, psychosocial adverse school and family experiences, lifestyle) showing significant effect sizes. Therefore, high SOC and self-esteem, school involvement, higher levels of physical activity would influence lower levels of depressive symptoms among adolescents. Exposure to negative acts at school and negative experiences in the family, psychosomatic health complaints, smoking would increase the probability of depressive symptoms among adolescents. Girls are more prone to depression as compared to boys.

## 7. Recommendations

The present study expands the existing literature and supplements today’s knowledge on the relationship between personal, psychosocial and lifestyle factors and depressive symptoms among adolescents. These findings extend our insight into the mechanisms underlying the associations among SOC, self-esteem, psychosocial (exposure to negative acts at school, adverse family environment, psychosomatic health complaints) and lifestyle factors (physical activity, smoking, alcohol) and depressive symptoms among adolescents. Those theoretical implications should be extended to adolescent resilience promotion programs and should focus on improving support in a school and family context, developing individual self-esteem and strengthening SOC. Involvement in physical activity should include participating in a social environment, as this could be an important aspect of the positive effect of exercise on mental health. More effective anti-smoking strategies should be implemented in school health promotion programs.

The review of current child and adolescent depression prevention programs revealed that the vast majority coincide in adopting a cognitive-behavioral approach, with contents including social skills and problem-solving training, emotional education, cognitive restructuring, and strategies for coping with anxiety and depression [44]. Enhancing youth engagement in these programs is necessary. However, if we accept that depression is multifactorial and that risk and protection factors may be found not only in the school environment but also in the family and social contexts, prevention should also be multifactorial.

## Figures and Tables

**Table 1:** Correlations of study variables.

	1	2	3	4	5	6	7	8	9	10	11
1. Depressive symptoms	1	−0.094 **	−0.612 **	0.530 **	0.405 **	0.467 **	0.578 **	−0.442 **	0.216 **	0.082 **	0.078 **
2. Gender	−0.094 **	1	−0.008	0.036	−0.069 **	0.03	−0.086 **	−0.067 **	−0.300 **	0.031	0.042 *
3. Sense of coherence	−0.612 **	−0.008	1	−0.346 **	−0.275 **	−0.459 **	−0.433 **	0.255 **	−0.101 **	−0.139 **	−0.143 **
4. Negative acts	0.530 **	0.036	−0.346 **	1	0.332 **	0.247 **	0.297 **	−0.321 **	0.074 **	0.041	0.031
5. FSAV	0.405 **	−0.069 **	−0.275 **	0.332 **	1	0.193 **	0.257 **	−0.164 **	0.119 **	0.208 **	0.171 **
6. Self-esteem	0.467 **	0.03	−0.459 **	0.247 **	0.193 **	1	0.256 **	−0.217 **	.110 **	−0.014	0.016
7. Psychosomatic health complaints	0.578 **	−0.086 **	−0.433 **	0.297 **	0.257 **	0.256 **	1	−0.215 **	0.068 **	0.156 **	0.178 **
8. School involvement	−0.442 **	−0.067 **	0.255 **	−0.321 **	−0.164 **	−0.217 **	−0.215 **	1	−0.110 **	−0.003	−0.050 *
9. Physical activity	0.216 **	−0.300 **	−0.101 **	0.074 **	0.119 **	0.110 **	0.068 **	−0.110 **	1	0.056 **	0.080 **
10. Alcohol	0.082 **	0.031	−0.139 **	0.041	0.208 **	−0.014	0.156 **	−0.003	0.056 **	1	0.453 **
11. Smoking	0.078 **	0.042 *	−0.143 **	0.031	0.171 **	0.016	0.178 **	−0.050 *	0.080 **	0.453 **	1

Notes. FSAV—Family stress and violence. * *p* < 0.05, ** *p* < 0.01.

**Table 2:** Predictors of depressive symptoms among adolescents (simple and hierarchical linear regression analysis).

	Simple Linear Regression		Hierarchical Regression			
Variable	Standardized Beta	R Square	Standardized Beta			
			Model I	Model II	Model III	Model IV
Sense of coherence	−0.605 *	0.366	−0.605 *	−0.453 *	−0.257 *	−0.263 *
Negative acts at school	0.519 *	0.269		0.306 *	0.205 *	0.204 *
Family stress and violence	0.396 *	0.157		0.172 *	0.124 *	0.122 *
Self-esteem	0.458 *	0.210			0.154 *	0.144 *
PHC	0.575 *	0.330			0.296 *	0.297 *
School involvement	−0.434 *	0.188			−0.187 *	−0.185 *
Gender	−0.093 *	0.009				−0.056 *
Physical activity	0.212 *	0.045				0.088 *
Smoking	0.077 *	0.006				0.050 *
Alcohol	0.081 *	0.007				0.014
R square			0.366	0.501	0.633	0.649
R square change				0.135 *	0.132 *	0.017*

Notes. PHC—Psychosomatic health complaints. * *p* < 0.001.

## Data Availability

Data generated and analyzed during the current study are not publicly available due to ethical reasons but are available for corresponding authors.
